# Cellular Therapy for Repair of Cardiac Damage after Acute Myocardial Infarction

**DOI:** 10.1155/2009/906507

**Published:** 2009-03-29

**Authors:** Matthew M. Cook, Katarina Kollar, Gary P. Brooke, Kerry Atkinson

**Affiliations:** ^1^Adult Stem Cell Laboratory, Mater Medical Research Institute, South Brisbane, QLD 4101, Australia; ^2^School of Medicine, University of Queensland, St Lucia, QLD 4072, Australia

## Abstract

Cardiovascular diseases, particularly acute myocardial infarction, are the leading causes of death worldwide. Important advances have been made in the secondary treatment for cardiovascular diseases such as heart transplantation and medical and surgical therapies. Although these therapies alleviate symptoms, and may even improve survival, none can reverse the disease process and directly repair the lasting damage. Thus, the cure of cardiovascular diseases remains a major unmet medical need. Recently, cellular therapy has been proposed as a candidate treatment for this. Many stem and progenitor cell populations have each been suggested as a potential basis for such therapy. This review assesses some of the more notable exogenous adult cell candidates and provides insights into the mechanisms by which they may mediate improvement in cardiac function following acute myocardial infarction. Research into the cellular therapy field is of great importance for the further planning of clinical trials for cardiac cellular myoplasty.

## 1. Introduction

Cardiovascular disease (CVD) is a broad term referring to all diseases that involve the heart and/or blood vessels. CVD is the leading cause of death worldwide, estimated at causing 17.5 million deaths in 2005. Of these an estimated 7.6 million were due to ischaemic heart disease (IHD) which is a subset of CVD and is characterised by occlusion of a coronary artery causing decreased blood flow and deprivation of oxygen and nutrients to the high energy-requiring cardiomyocytes in the myocardium [1]. This situation is also known as acute myocardial infarction (AMI) or more commonly a heart attack. While this may be an acute or transient condition (i.e., the heart is reperfused after a temporary occlusion), the ischemic damage due to loss of blood flow causes significant cardiomyocyte death and the subsequent irreversible formation of a fibrotic scar [2]. This, in turn, leads to dyskinesis of the ventricular wall, diminished heart function, and chronic heart failure (CHF).

Within the last 10–15 years, pharmaceutical therapies (aspirin, angiotensin converting enzyme-inhibitors, *β*-blockers), percutaneous coronary interventions, coronary artery bypass graft surgery, left ventricular assist devices and biventricular pacing have made a significant impact on the cardiovascular disease burden. Heart transplantation is also well established, but the need for long-term immune suppression and the chronic donor organ shortages suggest that it is unlikely to evolve into a viable definitive treatment for the majority of persons with cardiac disease. Although these therapies ameliorate symptoms, and may even improve survival, none can claim to directly reverse the disease process itself.

With recent advances in medicine and associated technologies, a number of novel therapies for the repair of the myocardium following ischaemia have been suggested. The most prominent of these is the use of cells to repair existing cardiac damage with bone marrow being the commonest source due to its readily accessible nature and autologous sourcing. Bone marrow mononuclear cells (BMCs), peripheral blood stem cells (PBSCs), mesenchymal stem cells (MSCs), and endothelial progenitor cells (EPCs) have been investigated in animal and human studies. However, many of these cell populations have been incompletely characterised and are thought to be a mixture of several related subpopulations. At present, BMCs are the most common source for cell-based therapy and, due to the 40 years of experience with bone marrow transplants used for treating haematological diseases, they have rapidly been applied to clinical trials in severe cardiac disease. Although the first phase I/II controlled clinical trials using BMCs to improve cardiac function after AMI were recently published (with variable results) [3, 4], it is still unclear as to which bone marrow cell population contributed to the repair of cardiac muscle damaged by ischaemia in these trials. This review will clarify the potential of purified adult cell populations to repair the damaged myocardium and outline several possible mechanisms of repair.

## 2. Stem/Progenitor Cell Populations

**Figure 1:**
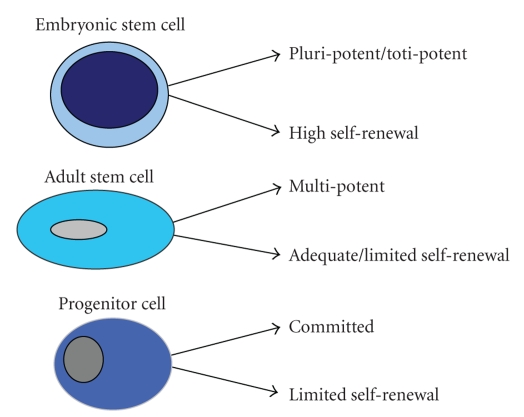
Differentiation and self-renewal potential of embryonic stem cells, adult stem cells, and progenitor cells.

Stem cells are defined by two key characteristics. Firstly, they must be able to self-renew in that they can go through many cell division cycles whilst remaining in an unchanged and undifferentiated state. Their second property is the capacity to differentiate into multiple specialised cell types [5], and the potency of stem cells is often defined by their potential to differentiate. At one end of the spectrum stem cells are able to differentiate into specialised mature cells from all three germ layers. These are termed pluripotent stem cells with embryonic stem cells (derived from blastocyst of a developing embryo) as the prime example. Alternately, multipotent stem cells are restricted to differentiating into certain closely related lineages often of the same germ layer [5, 6]. Adult (or somatic) stem cells are derived from postnatal or mature tissues and are primarily multipotent. However, some subsets have shown plasticity across multiple germ layers [7, 8]. Some adult stem cells have a limited capacity for self-renewal and may thus be better classified as adult progenitor cells. Although progenitor cells are also defined by the properties of self-renewal and multipotency, these are observed to a lesser extent compared to pluripotent stem cells. Hence, progenitor cells are more committed to specific lineage differentiation than stem cells (Figure 1).

### 2.1. Mesenchymal Stem Cells (MSCs)

**Figure 2:**
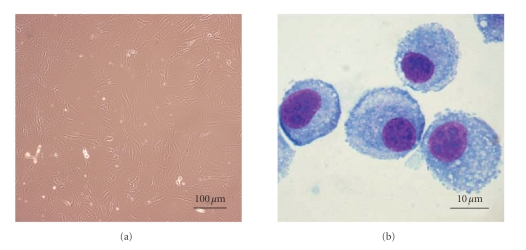
Mesenchymal stem cell morphology by light microscopy. (a) MSCs culture (×100), (b) Cytospin and Giemsa stained (×1000).

MSCs were first recognised by Friedenstein et al., who identified a plastic adherent, fibroblast-like population (Figure 2) that could regenerate rudiments of normal bone in vivo [9–11]. MSCs were initially identified within the stroma of the bone marrow and were subsequently found to provide support for haematopoiesis by secreting many colony-stimulating factors and growth factors important in the proliferation, differentiation and survival of haematopoietic cells [12–15]. Although a majority of the literature is concerned with bone marrow derived MSCs, they have also, in more recent times, been isolated from other organs including placenta, adipose, cord-blood, and liver [8, 16–21]. Recently, they have been shown to be ubiquitous since they have been shown to be an integral part of the composition of endothelium (perivascular cells or pericytes) [22, 23].

Although no single marker specific for MSCs has yet been described, they are characteristically negative for typical haematopoietic lineage antigens such as CD45, CD34, and CD14 and positive for CD44, CD73, CD90, CD105, CD166, and Stro-1 [24–29]. MSCs also produce a large number of growth factors and cytokines, including vascular endothelial growth factor (VEGF) [13]. This suggests MSCs involvement in paracrine mechanisms. However, the current hallmark for MSCs is their ability to differentiate into mature cell populations of the mesodermal lineage such as bone, cartilage, tendon, muscle, and adipose tissue [25, 28, 30, 31]. Interestingly, some groups have also shown MSCs to have plasticity beyond the mesodermal lineage with the ability to differentiate into neurons and astrocytes (ectodermal) as well as hepatocytes (endodermal) [7, 8].

MSCs also appear to have a significant advantage for cell therapy in that they are immunologically privileged and even in large outbred animals can be transplanted across major histocompatibility (MHC) barriers without the need for immune suppression [32–34]. This has important implications in that MSCs can be taken from a healthy unrelated donor and cryopreserved ready for use in patients with a wide variety of pathologies [35]. In addition to this relative lack of immunogenicity, MSCs are actively suppressive of T cell function [36, 37]. This immune suppressive capability has been successfully exploited in the clinic where unrelated, MHC-unmatched or mismatched MSCs have been used to treat patients with acute graft-versus-host disease (GVHD) [33, 38, 39].

MSCs show promise as a cellular therapeutic agent due to their ease of expansion, immuno-privileged status, and ability to self-renew and to differentiate across multiple mature cell lineages. Because of their multipotency, they have been investigated in cardiovascular disease [40, 41], neurological disease [42], osteogenesis imperfecta [43–45], osteoarthritis [46], GVHD [33, 39], and liver fibrosis [47]. Controversially, MSCs have been induced to differentiate into cardiomyocytes in mice [48] and humans [41], and several studies have shown improved myocardial function after myocardial ischaemia in rodents and pigs [40, 41]. This indicates their potential for treatment of damage caused by ischemic myocardial infarction in human. Finally, there are some reports that MSCs migrate preferentially to sites of inflammation as opposed to the bone marrow, as has been reported in some studies to be the case in unperturbed animals [26, 28].

### 2.2. Haematopoietic Stem/Progenitor Cells (HSCs/HPCs)

HSCs/HPCs are the best characterised adult stem cell and are the only stem or progenitor cells that are in routine clinical use today. HSCs normally reside in the bone marrow and are responsible for making all blood cell types and thus continually reconstituting the haematopoietic and immune systems [49, 50]. These cells are used clinically in bone marrow transplants to treat a number of blood disorders including leukaemia, aplastic anaemia, and severe combined immunodeficiency [51, 52]. The functional hallmark of a true HSCs is the in vivo ability to reconstitute all blood lineages (from a single cell) following otherwise lethal total body irradiation (TBI).

In the human, HSCs/HPCs are identified by cell surface expression of CD34, a cell surface glycoprotein [49, 53, 54]. However, isolation of CD34^+^ cells from umbilical cord blood, bone marrow, or peripheral blood aphaeresis product leads to a relatively heterogeneous population, while true HSCs, as defined by single-cell repopulation capacity, represent less then 0.1% of CD34^+^ enriched cells [55]. Thus, a CD34 enriched population is often referred to as an HPC population containing lineage-committed cells as well as HSCs. Although CD34 is also expressed in the mouse, the regulation of the CD34 gene is different to that in the human [53]. Thus, it is not a reliable HPC marker in the mouse. Furthermore, it has been shown that CD34^−/low^ murine HSCs can reconstitute the haematopoietic system following TBI [54].

**Figure 3:**
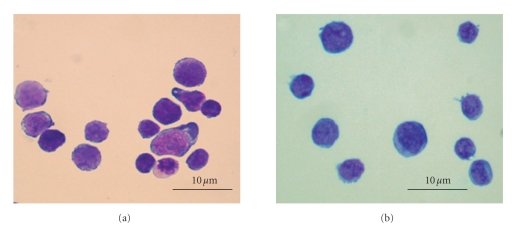
Morphology of (a) haematopoietic progenitor cells displaying a heterogeneous morphology and (b) the more purified LSK showing a more homogeneous morphology (light microscopy ×1000).

Murine studies of HSCs often use the following markers: c-kit^+^, CD45^+^, and lineage^−/low^. Lineage^−/low^ status is commonly defined as negativity for CD5 (T cells), CD11b (myeloid cells), CD45R (B cells), Gr-1 (granulocytes), and Ter119 (erythrocytes). This population can be further purified with the addition of the Sca-1^+^ marker to the panel and these HSCs are termed LSK cells (Lineage/Sca-1/c-kit) (Figure 3). However, these cells still represent a heterogeneous population with approximately one in ten having the ability to repopulate the haematopoietic system [56]. More recently, the signal lymphocyte activating molecule (SLAM) receptors (CD48, CD150, and CD244) have been used to further enrich the LSK population to derive a more primitive HSC population [57–59]. In humans, earlier HSCs are identified by the phenotypes CD34^+^ CD90^+^ or CD34^+^ CD38^−^ [60].

HPCs migrate preferentially to the bone marrow in healthy animals and the molecular mechanisms of this migration are well described, including rolling and tethering of HPCs on bone marrow endothelium, followed by their arrest and firm adhesion to the endothelium [61–63]. It is not known if intravenously injected HPCs migrate preferentially to acutely inflamed tissue.

It is debated as to whether bone marrow cells enriched for HPCs participate in the repair of cardiomyocytes following infarction. Orlic et al. (2001) and Rota et al. (2007) have shown that bone marrow cells enriched for c-kit are capable of differentiating into cardiomyocytes [64, 65]. However, these c-kit^+^ enriched populations were heterogeneous and may have contained stromal cell populations. Furthermore, HPCs have been observed to differentiate into skeletal muscle fibres during muscle regeneration [66]. Conversely, it has also been shown that HPCs do not differentiate into cardiomyocytes following infarction and rather take on a mature haematopoietic fate which may, in turn, give rise to differentiated haematopoietic cells, responsible for the inflammatory wound healing process [67, 68]. Various other actions of HPCs on injured tissue have been proposed including secretion of cytokines and chemokines, inhibition of apoptosis, and suppression of immune reactions [69, 70].

### 2.3. Endothelial Progenitor Cells (EPCs)

Historically, neoangiogenesis (the formation of new blood vessels) was thought to occur by the proliferation of existing endothelial cells. However, in 1997 Asahara et al. discovered “putative progenitor endothelial cells” in adult peripheral blood [71]. In the last 10 years, this discovery has been confirmed and it is now commonly accepted that EPCs play an essential role in adult blood vessel formation, endothelial repair, and endothelial homeostasis [72, 73]. These postnatal cells have been extensively explored for their innate capacity to contribute to angiogenesis in both pathological and unperturbed states. Like MSCs and HPCs, EPCs can also be derived from the bone marrow. They circulate in peripheral blood in a very low numbers, but can be mobilised into the blood using molecules such as granulocyte colony-stimulating factor (G-CSF) [74].

EPCs derived from adult human peripheral blood were initially characterised by expression of both CD34^+^ and Flk-1^+^ [71]. Flk-1 is also known as vascular endothelial growth factor receptor (VEGFR)-2 in mice and kinase insert domain receptor (KDR) in humans. Along with vascular endothelial growth factor receptor-1 (VEGFR-1, also known as Flt-1), VEGFR-2 serves to mediate the actions of VEGF, which is recognised as an essential regulator of angiogenesis [74, 75]. VEGFR-2 expression is considered to be a marker of progenitor cell commitment to the endothelial lineage and is now typically used in conjunction with other antigens, such as vascular-endothelium (VE)-cadherin (CD144) and CD31 (also known as platelet/endothelial cell adhesion molecule-1 or PECAM-1) to identify putative angioblasts or EPCs [76, 77]. Another key marker is CD133 (prominin-1) and, like CD34, it is present on both HPCs and cells that exhibit a potent blood-vessel forming capacity [78, 79]. In the developing embryo, EPCs and HSCs arise from a common precursor called the haemangioblast [80, 81]. Hence, a majority of markers used to identify EPCs are also prevalent on haematopoietic progenitors. Although these markers prove useful in identifying populations enriched for the angioblast or EPCs, it should be recognised that defining hierarchical relationships is far from currently agreed upon [77, 82].

**Figure 4:**
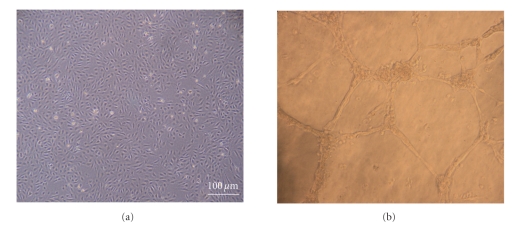
Endothelial progenitor cells derived from human umbilical cord blood (a) exhibit classic cobble-stone morphology (light microscopy ×40) and (b) form tube-like structures when seeded on Matrigel basement membrane matrix (light microscopy ×100).

It is now more common for EPCs to be isolated from peripheral blood, bone marrow, or umbilical cord blood (UCB) based on their morphologic and adherent characteristics when cultured on fibronectin or collagen-coated plates in the presence of appropriate growth media and supplemental angiogenic differentiation factors. These factors include VEGF, fibroblast growth factor-2 (FGF-2), insulin-like growth factor-1 (IGF-1), and epidermal growth factor (EGF) [75, 76, 83, 84]. The majority of cells that appear in early stages of culture (within the first 15 days) are thought to have originated from a CD14^+^ (macrophage/monocyte) subpopulation of mononuclear cells. These cells are often referred to as early-outgrowth endothelial progenitor cells (EO-EPCs) [75, 83, 85]. Conversely, late-outgrowth EPCs (LO-EPCs) do not appear in culture for 2-3 weeks and exhibit the “classic endothelial” phenotype with contact inhibition and cobblestone monolayer morphology and the ability to form in vitro tube-like structures when seeded on Matrigel (Figure 4). These LO-EPCs also show exponential growth kinetics and a capacity for ex vivo expansion [75, 77, 83, 85, 86].

It has been extensively shown that EPCs are associated with neoangiogenesis following tissue injury in animal models of hind-limb ischaemia and myocardial infarction [4, 84, 87, 88]. Conversely, however, administration of EPCs into human patients with CVD is yet to show efficacy with regard to vessel formation, even though these patients had a better clinical outcome [89]. This observation suggests that improvement may have been due to, at least in part, a paracrine function.

### 2.4. Non-Stem/Progenitor Cell Populations

It is well established that monocytes/macrophages play an important role in angiogenesis, first shown in 1977 [90]. This occurs by the release of tumour necrosis factor-*α* (TNF-*α*), thrombospondin, and angiogenic factors such as VEGF, angiopoietin, and matrix metalloproteinases (MMPs) [91–93]. Cells of the macrophage lineage play a major role in the innate immune response and contribute to wound healing, tissue repair, and bone remodeling [94]. Any disturbance of tissue normality such as infection, aberrant cell turnover, or tissue damage leads to an inflammatory response and a rapid recruitment of macrophages. During inflammation monocytes and macrophages phagocytose foreign particles (cellular debris or pathogens) and stimulate lymphocytes and other immune cells to respond to the pathogen by release of a variety of cytokines and chemoattractants that can modulate the migration of circulating cells and their adhesion to local endothelial cells [2, 91, 93, 95].

A novel lineage of monocytes has recently been isolated that is thought to play a key role in the revascularisation process. These cells are isolated from mice as CD11b^+^ monocytes (or from human as CD14^+^ and CD16^−^) and express the angiogenic marker Tie-2 (tunica internal endothelial cell kinase-2) [96–99]. Tie-2 is a receptor tyrosine kinase expressed principally on vascular endothelium and is also expressed on HPCs and EPCs. Its ligands are the angiopoietin growth factors that promote the growth of new blood vessels and are involved with migration of Tie-2-expressing cells to sites of inflammation [96–98]. Tie-2 has also been hypothesised to be involved in the attenuation of proinflammatory mediators.

**Figure 5:**
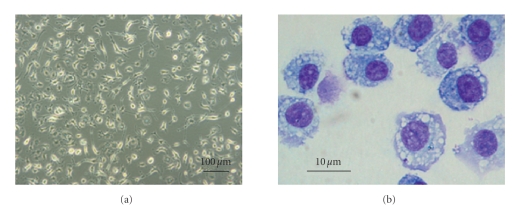
Analysis of Tie-2 expressing monocytes by (a) culture morphology (light microscopy ×100) and (b) Giemsa staining (light microscopy ×1000).

Tie-2 expressing monocytes (TEMs) have been isolated from the bone marrow of mice by plastic adherence and stimulation with macrophage colony-stimulating factor (M-CSF or CSF-1). These cells have a large nucleus surrounded by a cytoplasm with an abundance of vacuoles (Figure 5). They have also been identified in the peripheral blood of mice [96] and from human PBMCs [98, 99]. However, the literature on this subject has been focused on how TEMs may contribute to tumour-associated angiogenesis and thus these cells have often been identified and isolated from various mouse and human tumours. It is thought that TEMs may contribute to tumour angiogenesis by providing paracrine support to nascent blood vessels and by the sequestering of endothelial cells. TEMs produce proangiogenic factors and, when injected in Matrigel matrix plugs implanted under the skin of rodents, promote robust angiogenesis whilst not forming new blood vessels themselves. These data suggest that their recruitment to the site of ischaemic injury is sufficient to support revascularisation [96, 98, 100].

Monocytes are attracted to the myocardium by overexpression of monocyte chemoattractant protein-1 (MCP-1), where there is evidence that they form CD31-negative (PECAM) tunnels. Monocytes and macrophages “drill tunnels” using matrix metalloproteinases (MMPs). However it is yet to be demonstrated whether these become new vessels [100–102]. It has been proposed that neoangiogenesis occurs via the contribution of monocytes, macrophages, and circulating EPCs.

## 3. Possible Mechanisms of Tissue Repair by Cells

### 3.1. Differentiation into Cardiomyocytes and Cell Fusion

Stem cells may have the potential to replace necrotic myocardium, form new cardiomyocytes, and restore cardiac function after AMI [5, 103]. Cardiomyocyte differentiation in vitro has been shown with embryonic stem cells [104], HPCs [64, 65], MSCs [41], and EPCs [105]. Few studies, however, have been able to replicate this phenomenon in vivo and significant cell numbers have failed to differentiate in the myocardium [67, 106]. Although these studies showed a lack of physical repair of the myocardium, there was still, in most cases, an improvement in cardiac function. These data suggest that there may be other mechanisms mediating functional improvement. One alternative to differentiation is the occurrence of cell fusion to support cardiomyogenesis. However, the occurrence of this is also rare [107–109].

### 3.2. Paracrine Function

Stem cells secrete many soluble factors that may directly or indirectly have reparative qualities. In cardiac ischaemia, these factors may signal through pathways that act to promote angiogenesis, decrease apoptosis, increase the efficiency of cardiomyocyte metabolism, or modulate inotrophy (formation of fibrous scar) [2]. There is evidence of bone marrow mononuclear cell-conditioned media showing reparative features in preclinical models of AMI [110–113]. This conditioned media contained a variety of cytokines including VEGF, interleukin-1 (IL-1), placental-derived growth factor (PDGF), IGF-1 and MCP-1. It has also been shown that these factors were significantly upregulated when the cells are cultured in a hypoxic state [114]. Transfusion of this media led to increased capillary density, decreased infarct size, and improvement of cardiac function following myocardial infarction [110, 112]. Whilst transfusion of the conditioned media is potentially seen as a useful therapeutic agent, the need for the donor cells to be transfused was still apparent as it was shown that some functional reparative mechanisms only occurred when the bone marrow derived cells were present. There is also a need for such a pool of cytokines to be constantly replenished, a function that can be performed by constant transfusion of conditioned media or a single transfusion of a cellular “cytokine factory”.

### 3.3. Vascular Remodeling

Interventions that increase the perfusion to areas of restricted blood flow may cause salvage of border-zone cardiomyocytes as well as partial reversal of pathological myocardial remodeling. Increased vascularisation may occur via two pathways. Firstly, it may occur by angiogenesis which is the formation of new branches of blood vessels from pre-existing vessels and thus an increase in perfusion. Secondly, it may occur by an increase in diameter of existing vessels causing an increase in local perfusion [104]. The success of both these events is determined by the pre-existence of a vascular network, activation of the endothelium by fluid pressure stress, invasion of bone marrow derived cells, and proliferation of endothelial and smooth muscle cells [115]. Cell therapy may act in a paracrine mechanism to increase the production of proangiogenic cytokines (as mentioned above) and stimulate host cells to remodel the existing vasculature by either forming new branches or converting an arteriole into an artery. Alternately, donor cells may physically embed in the ischaemic tissue and become new vessel branches.

Various studies have shown revascularisation in animal models of hind-limb ischaemia and myocardial infarction with EPCs [87, 116–118], HPCs [64, 66], BMCs [112, 119], and MSCs [120]. One such study by Kocher et al. [81] showed that human mobilised CD34^+^ cells homed to the infarcted myocardium where they were incorporated into newly developed coronary circulation. However, similar to the differentiation process above, there is limited evidence showing that the cells themselves are directly or physically participating in this event [121]. This, once again, reinforces the potential value of the paracrine function of infused cells as they are often associated with an increase in capillary density in the infarcted region.

### 3.4. Cardiac Protection

Donor cell secretion of numerous cytokines is believed to attenuate the apoptotic state. For example, IGF-1 [122], hepatocyte growth factor (HGF) [123], and FGF [124] have all been shown to be cardioprotective molecules by attenuation of cardiomyocyte apoptosis.

Another form of cardiac protection is by modification of the immune response and this has subsequently been acknowledged as a potential therapeutic target [104]. This has been shown with MSCs, which suppress T-cell responses and thus have local immunosuppressive functionalities [32, 39]. However, cardiac protection through these mechanisms must be viewed with caution as many proinflammatory and proapoptotic molecules are also involved in angiogenic responses.

## 4. Conclusions

The field of regenerative medicine is quickly evolving and has been applied to many aspects of medicine including the treatment of CVD. Cellular therapy has been proposed as a candidate treatment for CVD. However, a majority of the clinical studies have used mixed populations of cells. Thus, it is still unclear as to which cell population contributed to the repair of cardiac muscle damaged by ischaemia in these trials. This review has outlined the characteristics of some notable purified cell populations that can be used to elucidate the possible roles and contributions of each cell type. Each cell type has its own advantages and limitations towards application for the treatment of CVD. This review has also described the potential mechanisms by which these cells mediate improvement in cardiac function following myocardial infarction. Possible mechanisms of cardiac cellular myoplasty include the generation of proangiogenic proteins, stimulation of new blood vessel formation, decrease in apoptosis of cardiac cells, decrease in pathological cardiac remodeling, differentiation into cardiomyocytes and cell fusion. Taking into consideration the various mechanisms of repair and the characteristics of each cell population, a cocktail of specific cell types may need to be considered for successful CVD cellular therapy.
